# Characterizing the Novel CRP, IP-10, and TRAIL Host-Response Protein Signature in Pediatric *Kingella kingae* Infections—A Retrospective Cohort Study

**DOI:** 10.3390/pediatric18050133

**Published:** 2026-10-02

**Authors:** Vered Nir, Dima Mreisat, Vered Schichter Konfino, Naama Kuchinski Cohen, Maanit Shapira, Milena Pitashny, Adi Klein

**Affiliations:** 1 Department of Pediatrics, Hillel Yaffe Medical Center, Hadera 3820302, Israel; 2 Ruth and Bruce Rappaport Faculty of Medicine, Technion—Israel Institute of Technology, Haifa 3200003, Israel; demamresat@gmail.com (D.M.);; 3 Laboratory Division, Hematology Laboratory, Hillel Yaffe Medical Center, Hadera 3820302, Israel; 4 Clinical Microbiology Laboratory, Hillel Yaffe Medical Center, Hadera 3820302, Israel

**Keywords:** *Kingella kingae*, inflammatory markers, IP-10, TRAIL, MeMed, pediatric infections

## Abstract

**Background**: *Kingella kingae* is a primary cause of pediatric osteoarticular infections. Diagnosis is challenging due to mild clinical presentations and fastidious culture growth, often mimicking viral illnesses. The MeMed BV™ assay measures C-reactive protein (CRP), interferon gamma-induced protein 10 (IP-10), and Tumor Necrosis Factor-Related Apoptosis-Inducing Ligand (TRAIL) to differentiate bacterial from viral infections, but its utility in *K. kingae* has not been evaluated. **Objectives**: To characterize the CRP, IP-10, and TRAIL profiles in pediatric patients with confirmed *K. kingae* infections compared to viral and classical bacterial cohorts. **Methods**: This retrospective study analyzed 19 children with microbiologically confirmed *K. kingae*. Controls included viral (*n* = 20), pneumococcal (*n* = 17) and Gram-negative (*n* = 7) cohorts. Serum concentrations of the three host-response proteins were compared. **Results**: The median age of the *K. kingae* cohort was 11.0 months (IQR, 9.0–12.0 months). Compared to viral controls, *K. kingae* elicited significantly lower IP-10 levels (median: 100.0 pg/mL [IQR, <100.0–399.0] vs. 632.5 pg/mL [IQR, 346.3–1375.8]; *p* = 0.004) and a downward trend in TRAIL levels (median: 75.0 pg/mL [IQR, 66.0–95.0] vs. 169.5 pg/mL [IQR, 76.5–205.0]; *p* = 0.066), aligning with a bacterial profile. Conversely, *K. kingae* induced significantly lower inflammatory and host-response markers than typical bacterial infections; its median CRP (18.8 mg/L [IQR, 10.0–50.0]) and MeMed BV™ score (28 [IQR, 11–61]) were markedly lower than those observed in the pneumococcal cohort (CRP: 94.0 mg/L [IQR, 39.5–182.5]; MeMed BV: 89 [IQR, 51–98.5]) and the alternative bacterial cohort (CRP: 106.0 mg/L [IQR, 32.0–250.0]; MeMed BV: 73.0 [IQR, 67.0–99.0]). **Conclusions**: *Kingella kingae* infections elicit a distinct host-response fingerprint characterized by mild CRP elevation, leukocytosis, and uniquely suppressed IP-10 levels. Utilizing this three-protein signature may help distinguish *K. kingae* from viral mimics, preventing diagnostic delays and optimizing antibiotic stewardship.

## 1. Introduction

*Kingella kingae* is a fastidious Gram-negative bacterium and a common component of the oropharyngeal flora in young children, with asymptomatic carriage rates reaching approximately 10% [1]. Over the past two decades, it has emerged as the primary etiology of invasive osteoarticular infections in children aged 6–48 months [2,3], accounting for up to 87% of cases in this age group [4]. This prevalence is particularly heightened during outbreaks of hand, foot, and mouth disease [5].

The organism was first characterized as a major pathogen in southern Israel, where an annual incidence of invasive disease of 27.4 per 100,000 children under 24 months was documented, and where *K. kingae* was shown to be the leading cause of septic arthritis in children under 2 years [3]. Subsequent European cohorts, notably from Geneva, Switzerland, found *K. kingae* responsible for 51% of confirmed pediatric osteoarticular infections, suggesting the organism has become the dominant pathogen across multiple regions where molecular diagnostics are used [6].

A major clinical challenge is that *K. kingae* infections typically present with mild systemic symptoms, low-grade fever, and only mildly elevated inflammatory markers [4]. Furthermore, the bacterium is notoriously difficult to isolate using standard culture techniques [1,7]. These factors frequently lead to diagnostic delays, increasing the risk of long-term complications such as growth plate damage [8]. Conversely, *K. kingae* can occasionally trigger highly aggressive disease, including endocarditis [9], which carries a case-fatality rate exceeding 10% [8,10]. Consequently, early and accurate differentiation of *K. kingae* from viral illnesses or typical bacterial pathogens is essential. Suspecting a Kingella infection will lead to starting antibiotic treatment in a child who would otherwise be suspected of having a viral infection, thus allowing for earlier antibiotic initiation.

The MeMed BV™ test is a novel, host-response immunological assay that measures C-reactive protein (CRP), interferon gamma-induced protein 10 (IP-10), and Tumor Necrosis Factor-Related Apoptosis-Inducing Ligand (TRAIL) to differentiate bacterial from viral etiologies [11]. IP-10 is a chemokine involved in inflammatory cell migration; its serum levels rise during bacterial infections in correlation with disease severity [12]. In contrast, TRAIL participates in neutrophil apoptosis to limit tissue damage, and its levels are characteristically down-regulated during bacterial infections compared to viral ones [13].

The MeMed BV™ is used increasingly as a routine exam in order to differentiate between viral and bacterial infections and therefore reduce unnecessary antibiotic use and to help hasten treatment for bacterial infections. It has been used in our center since 2017 as part of the routine laboratory workup for pediatric patients presenting with febrile illness.

While CRP kinetics in *K. kingae* infections are well-documented, the diagnostic utility of IP-10 and TRAIL host-responses has not yet been evaluated in this specific population. This study aims to characterize this novel three-protein host signature in children with confirmed *K. kingae* infections and compare them to viral and typical bacterial cohorts.

## 2. Methods

### 2.1. Study Design and Setting

This is a single-center retrospective, observational study. The study period spanned from January 2017 to December 2024. The study protocol was reviewed and approved by the Institutional Review Board (Helsinki Committee, approval #HYMC-0123-24). This study was prepared and reported in accordance with the Strengthening the Reporting of Observational Studies in Epidemiology (STROBE) guidelines for observational retrospective studies.

### 2.2. Participant Selection

We reviewed the electronic medical records of pediatric patients (aged 0–18 years) admitted with suspected invasive bacterial infections. The inclusion criterion for the study group was microbiological confirmation of *K. kingae* isolated from blood cultures or synovial fluid cultures. Blood and synovial fluid samples were collected using standard aseptic procedures and inoculated into the BD BACTEC™ pediatric blood culture system (BD, Becton, Dickinson and Company, Franklin Lakes, NJ, USA), and incubated for continued monitoring for 5 days according to the manufacturer’s instructions. Positive bottles were subcultured into standard media under aerobic and anaerobic conditions. Colonies suspected of Kingella were identified using matrix-assisted laser desorption/ionization time-of-flight mass spectrometry (MALDI-TOF MS) with the Bruker MALDI Biotyper Sirius system (Bruker Daltonics, Bremen, Germany). Spectral profiles were compared with the Bruker MALDI Biotyper reference database, and identification of *Kingella kingae* was reported at the species level when the manufacturer’s recommended identification score criteria were met. Demographic, clinical, and laboratory data were extracted from the electronic medical records by unblinded investigators.

Due to the exploratory nature of this study and the rarity of microbiologically confirmed *K. kingae*, a convenience sample consisting of all eligible patients during the study period was utilized.

To evaluate the diagnostic performance of the host-response signature, three control groups were established:

Viral Control Group: Patients presenting with acute febrile illness and a laboratory-confirmed viral pathogen (e.g., Influenza A/B, RSV, Adenovirus, or Enterovirus) via multiplex PCR or rapid antigen testing. To minimize bias, inclusion required a clinical course entirely consistent with a viral etiology, no antibiotic intervention, and complete recovery.

Pneumococcal Control Group: Patients with *Streptococcus pneumoniae* isolated from sterile sites (blood, cerebrospinal fluid, or pleural fluid), representing a classic invasive bacterial infection.

Gram-Negative Control Group: Patients with Gram-negative bacteria isolated from sterile sites, representing a different bacterial infection (e.g., *Escherichia coli*, *Salmonella*, *Acinetobacter*).

Patients with mixed infections (bacterial–viral co-detections) or known significant immunodeficiencies were excluded from all cohorts.

### 2.3. Laboratory Analysis and Host-Response Signature

Routine laboratory parameters, including White Blood Cell (WBC) count and Absolute Neutrophil Count (ANC), were recorded. All blood samples were collected prior to the administration of the first antibiotic dose. Serum concentrations of CRP, IP-10, and TRAIL were quantified using the MeMed BV™ (MeMed, Andover, MA, USA) assay according to the manufacturer’s instructions in real time.

### 2.4. Statistical Analysis

Data normality was formally assessed using the Shapiro–Wilk test. Because the continuous variables—including age, duration of hospitalization, and host-response biomarker profiles (CRP, IP-10, and TRAIL levels)—demonstrated a non-normal distribution, they were expressed as medians with interquartile ranges (IQR). Consequently, differences in continuous variables between the study groups were analyzed using non-parametric methods, specifically the Mann–Whitney U test for two-group comparisons or the Kruskal–Wallis test for multi-group analyses, as appropriate. Categorical variables were expressed as frequencies and percentages and compared across cohorts using the Chi-square test or Fisher’s exact test. A two-tailed *p*-value < 0.05 was considered statistically significant for all analyses. All statistical computing and modeling were conducted using IBM SPSS Statistics version 29.0 (IBM Corp., Armonk, NY, USA).

## 3. Results

A total of 19 patients with confirmed *K. kingae* infection were identified through blood cultures or synovial fluid culture. These were compared against 20 viral controls, 17 pneumococcal controls and 7 Gram-negative controls.

### 3.1. Demographic and Clinical Characteristics

The baseline demographic and clinical features of the *K. kingae* cohort are summarized in Table 1. The median age of the *K. kingae* group was 11 months (IQR, 9–12 months), and it included 7 males (37%) and 12 females (63%). Eighteen patients (95%) were previously healthy; one patient had a history of congenital cataracts.

All 19 patients presented with fever. Associated symptoms included aphthous stomatitis (*n* = 4, 21%), vomiting and diarrhea (*n* = 4, 21%), and cough (*n* = 3, 16%). Localized joint involvement was documented in 7 patients (37%): three presented with elbow pain/swelling, three with hip involvement, and one with redness and swelling of the thumb. In these 7 patients, *K. kingae* was isolated directly from synovial fluid, whereas the remaining 12 patients (63%) had positive blood cultures.

Echocardiograms were performed in 10 patients; 9 were normal, and one revealed mild aortic valve regurgitation. The median duration of hospitalization was 4.0 days (IQR, 3.0–6.0 days; range: 0–11 days). All patients received intravenous antibiotics targeted to *K. kingae* (Cefazolin, *n* = 6; Ceftriaxone, *n* = 6; Amoxicillin, *n* = 3; Cefuroxime, *n* = 2) followed by a transition to oral therapy. All patients were discharged home following clinical improvement and resolution of fever.

### 3.2. Cohort Comparisons

The median age differed significantly across the study groups. Patients in the *K. kingae* cohort had a median age of 11.0 months (IQR, 9.0–12.0 months), which was significantly older than those in the viral cohort (median age: 5.0 months; IQR, 2.1–7.5 months; *p* < 0.001) but significantly younger than those in the pneumococcal cohort (median age: 16.0 months; IQR, 12.0–24.0 months; *p* = 0.007). In comparison, the alternative bacterial cohort demonstrated a median age of 12.0 months (IQR, 6.0–31.0 months). No statistically significant differences in gender distribution were observed across the four groups (Table 1).

### 3.3. Laboratory and Inflammatory Markers

Initial laboratory outcomes and host-response profiles across the cohorts are detailed in Table 2. The median CRP level in the *K. kingae* group was 18.8 mg/L (IQR, 10.0–50.0 mg/L). While this reflected higher systemic inflammation compared to the viral group (median CRP: 12.5 mg/L; IQR, 6.0–19.8 mg/L), the difference did not achieve statistical significance. Conversely, classic invasive bacterial infections elicited profoundly higher inflammatory responses; patients in the pneumococcal group demonstrated a significantly higher median CRP level of 94.0 mg/L (IQR, 39.5–182.5 mg/L; *p* = 0.015) compared to the *K. kingae* cohort, and the alternative bacterial cohort similarly presented with an elevated median CRP of 106.0 mg/L (IQR, 32.0–250.0 mg/L).

Initial laboratory outcomes and host-response markers are detailed in Table 2. Quantitative analyses revealed distinct immunological profiles across the four cohorts.

The median hemoglobin (HB) level in the *K. kingae* group was 11.2 g/dL (IQR, 10.7–11.8 g/dL), which was significantly lower than that observed in the viral cohort (median HB: 12.1 g/dL; IQR, 11.5–12.5 g/dL; *p* = 0.012), but comparable to both the pneumococcal cohort (median HB: 11.3 g/dL; IQR, 9.8–12.4 g/dL; *p* = 0.86) and the alternative bacterial cohort (median HB: 11.0 g/dL; IQR, 9.4–12.0 g/dL). White Blood Cell (WBC) counts in the *K. kingae* group (median: 14.4 × 10^3^/µL; IQR, 11.2–18.9 × 10^3^/µL) were significantly elevated compared to the viral group (median: 4.8 × 10^3^/µL; IQR, 3.8–6.4 × 10^3^/µL; *p* < 0.001), while showing no statistically significant differences when compared to the pneumococcal group (median: 16.5 × 10^3^/µL; IQR, 12.3–21.5 × 10^3^/µL; *p* = 0.081) or the alternative bacterial cohort (median: 15.1 × 10^3^/µL; IQR, 10.8–19.4 × 10^3^/µL).

Regarding the novel host-response protein signature, distinct patterns emerged across the cohorts. The median CRP level was lower in the *K. kingae* cohort (18.8 mg/L; IQR, 10.0–50.0 mg/L) than in the pneumococcal cohort (94.0 mg/L; IQR, 39.5–182.5 mg/L; *p* = 0.015) and the alternative bacterial cohort (106.0 mg/L; IQR, 32.0–250.0 mg/L), representing a milder classical inflammatory response. Conversely, CRP levels in the *K. kingae* group were higher than in the viral group (12.5 mg/L; IQR, 6.0–19.8 mg/L), though this did not reach statistical significance (*p* = 0.14).

Importantly, serum IP-10 levels were significantly lower in the *K. kingae* group (median: 100.0 pg/mL; IQR, <100.0–399.0 pg/mL) compared to the viral cohort (median: 632.5 pg/mL; IQR, 346.3–1375.8 pg/mL; *p* = 0.004), while demonstrating no significant difference from the pneumococcal cohort (median: 207.0 pg/mL; IQR, <100.0–399.0 pg/mL; *p* = 0.28) or the alternative bacterial cohort (median: 223.0 pg/mL; IQR, 141.0–467.0 pg/mL). Lastly, median TRAIL levels in the *K. kingae* group (75.0 pg/mL; IQR, 66.0–95.0 pg/mL) demonstrated a downward trend compared to the viral cohort (169.5 pg/mL; IQR, 76.5–205.0 pg/mL), approaching marginal significance (*p* = 0.066), while being non-significantly higher than the pneumococcal cohort (38.0 pg/mL; IQR, 25.0–66.0 pg/mL; *p* = 0.13) and comparable to the alternative bacterial cohort (50.0 pg/mL; IQR, 23.0–71.0 pg/mL.

## 4. Discussion

The aim of this study was to characterize the novel host-response protein signature comprising CRP, IP-10, and TRAIL in pediatric patients with confirmed *K. kingae* infections. Our findings suggest that *K. kingae* elicits a host-response profile that appears to bridge the gap between classical bacterial pathogens (including *S. pneumoniae* and other bacterial species) and typical viral illnesses.

Diagnosing *K. kingae* remains a challenge in pediatrics due to its fastidious nature in culture media and its frequent presentation with mild systemic symptoms and low-grade fevers. In our cohort, the relatively mild clinical presentation was mirrored by routine inflammatory markers; the median CRP in the *K. kingae* group (18.8 mg/L [IQR, 10.0–50.0 mg/L]) was significantly lower than that of the pneumococcal group (94.0 mg/L [IQR, 39.5–182.5 mg/L]) and lower than the alternative bacterial cohort (106.0 mg/L [IQR, 32.0–250.0 mg/L]). This observation highlights why relying solely on traditional markers like CRP might complicate the clinical differentiation between *K. kingae* and self-limiting viral infections.

Our preliminary data suggest a potential role for IP-10 and TRAIL biomarkers in evaluating these patients. IP-10 is an interferon-gamma-induced chemokine that typically rises during viral replication and certain severe bacterial infections. In our cohort, *K. kingae* induced low IP-10 levels (median: 100.0 pg/mL [IQR, <100.0–399.0 pg/mL]), which were significantly lower than those observed in viral infections (median: 632.5 pg/mL [IQR, 346.3–1375.8 pg/mL]) but comparable to the pneumococcal (median: 207.0 pg/mL [IQR, <100.0–399.0 pg/mL]) and alternative bacterial cohorts (median: 223.0 pg/mL [IQR, 141.0–467.0 pg/mL]). This observed suppression of IP-10 in *K. kingae* infections could serve as a helpful parameter to consider when viral etiologies are suspected in a young child presenting with subtle acute febrile illness or early bone/joint complaints.

Furthermore, TRAIL showed a downward trend in the *K. kingae* group compared to the viral cohort (median: 75.0 pg/mL [IQR, 66.0–95.0 pg/mL] vs. 169.5 pg/mL [IQR, 76.5–205.0 pg/mL], *p* = 0.066), while remaining close to the suppressed levels seen in the pneumococcal (median: 38.0 pg/mL [IQR, 25.0–66.0 pg/mL]) and alternative bacterial groups (median: 50.0 pg/mL [IQR, 23.0–71.0 pg/mL]). Although the viral-to-Kingella comparison did not reach statistical significance, the biological trend appears consistent with a bacterial host-response. Additionally, WBC counts in the *K. kingae* group (median: 14.4 × 10^3^/µL [IQR, 11.2–18.9 × 10^3^/µL]) were higher than in the viral group (median: 4.8 × 10^3^/µL [IQR, 3.8–6.4 × 10^3^/µL]), suggesting that a localized or modest leukocytosis may still occur despite the low levels of acute-phase proteins and viral-induced chemokines.

## 5. Study Limitations

This study has several limitations. First, it was conducted retrospectively at a single medical center, resulting in a relatively small sample size (*n* = 19 for *K. kingae*). Second, significant baseline age variations existed between the cohorts, reflecting the natural epidemiological niches of these pathogens; however, this mismatch could introduce confounding biological factors related to immune system maturity. Future prospective, multi-center studies with larger, tightly age-matched cohorts are warranted to validate the precise diagnostic cut-offs of the MeMed BV™ score in *K. kingae* populations.

## 6. Conclusions

Our preliminary findings suggest that *Kingella kingae* infections exhibit a nuanced host-response profile characterized by mildly elevated CRP and relatively low IP-10 levels compared to viral infections, while presenting with significantly lower inflammatory scores and CRP than typical bacterial pathogens. Integrating this multi-protein signature into pediatric evaluation pathways may aid in distinguishing *K. kingae* from both viral mimics and classic bacterial etiologies, potentially helping to optimize early treatment stratification and support targeted antibiotic stewardship.

## Figures and Tables

**Table 1:** Demographic characteristics by group at presentation.

	*Kingella kingae* (*n* = 19)	Viral Controls (*n* = 20)	*Streptococcus pneumoniae* (*n* = 17)	Alternative Bacterial Cohort (*n* = 7)	*p*-Value
Age (months), Median [IQR]	11.0 [9.0–12.0]	5.0 [2.1–7.5]	16.0 [11.5–36.5]	12.0 [6.0–31.0]	*p*_1_ < 0.001 *p*_2_ = 0.007 *p*_3_ > 0.05
Gender, *n* (%)					
Male	7 (37%)	7 (35%)	9 (53%)	4 (57%)	*p*_1_ = 0.900 *p*_2_ = 0.330 *p*_3_ = 0.410
Female	12 (63%)	13 (65%)	8 (47%)	3 (43%)	

*p*_1_ represents *K. kingae* vs. viral controls; *p*_2_ represents *K. kingae* vs. *S. pneumoniae*; *p*_3_ represents *K. kingae* vs. alternative bacterial cohort.

**Table 2:** Comparison of laboratory findings between the three patient groups.

	*Kingella kingae* (*n* = 19)	Viral Controls (*n* = 20)	*Streptococcus pneumoniae* (*n* = 17)	Alternative Bacterial Cohort (*n* = 7)	*p*_1_-Value	*p*_2_-Value	*p*_3_-Value
Hemoglobin (g/dL)	11.2 [10.7–11.8]	12.1 [11.5–12.5]	11.3 [9.8–12.4]	11.0 [9.4–12.0]	0.012	0.860	0.640
WBC Count (×10^3^/μL)	14.4 [11.2–18.9]	4.8 [3.8–6.4]	16.5 [12.3–21.5]	15.1 [10.8–19.4]	<0.001	0.081	0.720
CRP (mg/L)	18.8 [10.0–50.0]	12.5 [6.0–19.8]	94.0 [39.5–182.5]	106.0 [32.0–250.0]	0.140	0.015	0.024
IP-10 (pg/mL)	100.0 [<100–399]	632.5 [346.3–1375.8]	207.0 [<100–399]	223.0 [141.0–467.0]	0.004	0.280	0.180
TRAIL (pg/mL)	75.0 [66.0–95.0]	169.5 [76.5–205.0]	38.0 [25.0–66.0]	50.0 [23.0–71.0]	0.066	0.130	0.210

CRP—C-reactive protein; IP-10—interferon gamma-induced protein 10; TRAIL—Tumor Necrosis Factor-Related Apoptosis-Inducing Ligand; WBC—White Blood Cell. *p*_1_ – (*K. kingae* vs. Viral), *p*_2_ – (*K. kingae* vs. *Pneumo*) *p*_3_ – (*K. kingae* vs. Alt. Bact)

## Data Availability

The datasets generated during this study are not publicly available due to patient confidentiality protocols but are available from the corresponding author on reasonable request.
